# Appraisals of the Bangladeshi Medicinal Plant *Calotropis gigantea* Used by Folk Medicine Practitioners in the Management of COVID-19: A Biochemical and Computational Approach

**DOI:** 10.3389/fmolb.2021.625391

**Published:** 2021-05-26

**Authors:** Mycal Dutta, Mohammad Nezam, Subrata Chowdhury, Ahmed Rakib, Arkajyoti Paul, Saad Ahmed Sami, Md. Zia Uddin, Md. Sohel Rana, Shahadat Hossain, Yunus Effendi, Rinaldi Idroes, Trina Tallei, Ali M. Alqahtani, Talha Bin Emran

**Affiliations:** ^1^ Department of Pharmacy, BGC Trust University Bangladesh, Chittagong, Bangladesh; ^2^ Department of Pharmacy, Jahangirnagar University, Dhaka, Bangladesh; ^3^ Department of Pharmacy, Faculty of Biological Sciences, University of Chittagong, Chittagong, Bangladesh; ^4^ Atomic Energy Centre, Chittagong, Bangladesh; ^5^ Department of Biology, Faculty of Science and Technology, Al-Azhar Indonesia University, Jakarta, Indonesia; ^6^ Department of Pharmacy, Faculty of Mathematics and Natural Sciences, Syiah Kuala University, Banda Aceh, Indonesia; ^7^ Department of Biology, Faculty of Mathematics and Natural Sciences, Sam Ratulangi University, Manado, Indonesia; ^8^ Department of Pharmacology, College of Pharmacy, King Khalid University, Abha, Saudi Arabia

**Keywords:** COVID-19, SARS-CoV-2, M^pro^, *in silico*, *Calotropis gigantea*, M^pro^ inhibitor

## Abstract

Severe acute respiratory syndrome coronavirus 2 (SARS-CoV-2) was first recognized in Wuhan in late 2019 and, since then, had spread globally, eventually culminating in the ongoing pandemic. As there is a lack of targeted therapeutics, there is certain opportunity for the scientific community to develop new drugs or vaccines against COVID-19 and so many synthetic bioactive compounds are undergoing clinical trials. In most of the countries, due to the broad therapeutic spectrum and minimal side effects, medicinal plants have been used widely throughout history as traditional healing remedy. Because of the unavailability of synthetic bioactive antiviral drugs, hence all possible efforts have been focused on the search for new drugs and alternative medicines from different herbal formulations. In recent times, it has been assured that the M^pro^, also called 3CL^pro^, is the SARS-CoV-2 main protease enzyme responsible for viral reproduction and thereby impeding the host’s immune response. As such, M^pro^ represents a highly specified target for drugs capable of inhibitory action against coronavirus disease 2019 (COVID-19). As there continue to be no clear options for the treatment of COVID-19, the identification of potential candidates has become a necessity. The present investigation focuses on the *in silico* pharmacological activity of *Calotropis gigantea*, a large shrub, as a potential option for COVID-19 M^pro^ inhibition and includes an ADME/T profile analysis of that ligand. For this study, with the help of gas chromatography–mass spectrometry analysis of *C. gigantea* methanolic leaf extract, a total of 30 bioactive compounds were selected. Our analyses unveiled the top four options that might turn out to be prospective anti–SARS-CoV-2 lead molecules; these warrant further exploration as well as possible application in processes of drug development to combat COVID-19.

## Introduction

Coronavirus disease 2019 (COVID-19) is an infectious disease triggered by infection with the severe acute respiratory syndrome coronavirus 2 (SARS-CoV-2) virus. In late 2019, cases of a pneumonia of a then-unknown origin began to appear in the capital of China’s Hubei province, Wuhan, later progressively spreading throughout the globe, resulting in the current COVID-19 pandemic (Hui et al., 2020). COVID-19 is not the first respiratory disease that has caused an epidemic in the last two decades; previously, outbreaks of Middle East respiratory syndrome (MERS) and severe acute respiratory syndrome (SARS), both caused by other coronaviruses, have occurred (de Wit et al., 2016). Coronaviruses are enveloped positive-sense single-stranded RNA genome (26–32 kb) viruses (Su et al., 2016). To date, four genera of coronaviruses have been identified: coronavirus genera α (alpha), coronavirus genera β (beta), coronavirus genera γ (gamma), and coronavirus genera δ (delta). Human coronaviruses (HCoVs) detected thus far include the HCoV-229E and NL63 in the α-coronavirus genus and MERS-CoV, SARS-CoV, HCoV-OC43, and HCoV-HKU1 in the β-coronavirus genus (Perlman and Netland, 2009). Following virus genome sequencing in December 2019 of five hospitalized patients in Wuhan with the mysterious pneumonia, it was divulged the existence of a formerly unknown β-CoV strain existed in all of them. This isolated neoteric β-CoV manifests an 88% degree of specification to the sequence of two bat-derived SARS-like coronaviruses, bat-SL-CoVZC45 and bat-SL-CoVZXC21, and an almost 50% degree of specification to the sequence of MERS-CoV (Lu et al., 2020). Liu *et al.*, recently determined that there is a presence of the main protease enzyme in SARS-CoV-2 (Liu et al., 2020) responsible for inhibiting SARS-CoV-2 replication. Therefore, targeting this specific enzyme may lead to potential therapeutic advantages in drug discovery and efficacy for managing this dangerous virus. Elsewhere, experiments with four drugs approved by the United States Food and Drug Administration, including praziquantel, perampanel, nelfinavir, and pitavastatin as potent inhibitors for SARS-CoV-2 M^pro^ enzyme, are ongoing (Yusuf et al., 1994). Recently, researchers are also reviewing medicinal plants for possible efficacy in the context of SARS-CoV-2 infection (Khaerunnisa et al., 2020).

Having relatively less non-toxic property, synthetic bioactive natural compounds have diverse bioavailability. Various phytoconstituents like phenols, steroids, flavonoid molecules are present in the natural bioactive compounds and also they have been reported and screened for their possible therapeutic effects against various viral diseases. Certain natural compounds such as digitoxigenin, crocin, withanone, β-eudesmol, caffeic acid phenethyl ester and other various bioactive molecules have also been reported to interact with SARS-CoV-2 main protease (Tripathi et al., 2020). Traditional medicine employs a holistic approach to the prevention of disease and act as a source of many components with a high therapeutic value. Globally, many studies have been conducted on these herbs and revealing unique active constituents that might activate the innate immune system through the stimulation of macrophages and lymphocytes and modulation of the cytokine profile also (Tiwari et al., 2018). The potent therapeutics that might be effective to combat SARS-CoV-2 infection include virus binding molecules, helicase inhibitors molecules or inhibitors targeting particular enzymes implicated in replication and transcription process of the virus, protease inhibitors of host cells, endocytosis inhibitors, short interfering RNA (siRNA), neutralizing antibodies and natural drugs or medicines (Dhama et al., 2020).

Various kinds of secondary metabolites such glycosides, alkaloids, tannins, lignins and terpenes are present abundantly in various folklore medicinal plants and, thus, many studies have already explored the use of these plants in the treatment of various infections and diseases with the help of these secondary metabolites. Different extractable biochemical and bioactive compounds are contained in traditional medicinal plants might cure or prevent several viral diseases and infections by targeting certain specific sites of viruses. Despite the healing histories or scientific evidences, the use of medicinal plants and natural products as prophylactics, therapeutics and their multiple beneficial applications have gained strong momentum over the last few decades (Dhama et al., 2018; Brendler et al., 2020).

Henceforth, compound isolation and subsequent further investigation for several pharmacological activities has been established as a crucial process in the areas of drug design and discovery of novel drugs as well. The present study considered some isolated phytochemicals from a medicinal plant *Calotropis* *gigantea*, a member of the Apocynaceae family, for the treatment of COVID-19 using computational biology techniques. *C. gigantea*, frequently called giant milkweed or crown flower, is a large shrub or small tree native to Bangladesh, India, Malaysia, Southern China, and Indonesia. The plant is also locally known as Gulancha. These large shrubs have also been extensively cultivated in tropical areas around the world and typically grow to eight to 15 feet tall.

As calotropin offers potent bioactivity, *C. gigantea* has been used by the folk practitioners in India for many years for a variety of purposes. In Ayurveda, the root and leaf of *Calotropis procera* have been being used in managing asthma and breathing problems such as shortness of breath. The bark of *C. procera* is also being applied in other types of diseases such as spleen and liver conditions. This plant has been reported to be effective as well in treating skin, circulatory, digestive, and neurological issues and was also used previously to treat fevers, nausea, vomiting, and diarrhea. However, these reports are usually based on folklore uses and, therefore, more scientific investigations are necessary to affirm the rational and clinical usefulness of the leaves, latex, and bark of this family. Moreover, recent research has suggested the use of calotropin as a contraceptive and as a promising medication for cancer patients (Wang et al., 2009). In one study, the extracts of *C. gigantea* appeared to be strongly cytotoxic against non–small-cell lung carcinoma (A549), hepatocellular carcinoma (Hep G2), and colon carcinoma (HCT 116). In other research, leaf extracts of this plant have also shown promising activities as cancer medications (Jacinto et al., 2011). One species of the *Calotropis* genus, *C. procera*, displayed potent anti–human immunodeficiency virus 1 activity when the aqueous crude extract of the leaves was analyzed (Rakshit et al., 2010). An ethanolic extract of *C. gigantea* exhibited activity against herpes simplex type 1 viruses and vesicular stomatitis viruses (Ali et al., 1996). A novel lignan glycoside, (+)-pinoresinol 4-O-[6″-O-vanilloyl]-β-d-glucopyranoside, isolated from the latex of *C. gigantea* exhibited anti-influenza activity in an *in vitro* study (Parhira et al., 2014). Meanwhile, several other research works have suggested the protease inhibitory effect of *C. gigantea*.

In alignment with these previous findings, we speculate that the phytochemicals obtained from *C. gigantea* might be able to impede the main protease enzyme of SARS-CoV-2 (Kaladhar et al., 2013; Gupta and Chaphalkar, 2016). Through the process of gas chromatography–mass spectrophotometry (GC-MS), several compounds have been isolated for molecular docking. Molecular docking studies are of terrific significance in establishing plans for the layout of new drug substances; specifically, their strategic aim is to explore the binding affinity and the mode of binding of a small molecule within the specific binding sites of the receptor target of interest (Chikhale et al., 2020). This methodology is based on the prediction of native ligands that pose or bind with the proper receptor binding site, i.e., to find the experimental ligand geometry associated with physicochemical molecular interactions. The aim of this study was to screen out potent bioactive ligands from the GC-MS compound list of *C. gigantea* that could be probable inhibitors of M^pro^.

## Materials and Methods

### GC-MS Analysis

The methanolic leaf extract of *C. gigantea* was inspected in a mass spectrometer (TQ8040; Shimadzu Corporation, Kyoto, Japan) with assistance from of electron-impact ionization method and a gas chromatograph (GC-17A; Shimadzu Corporation) fused with a silica capillary column made by silica with the specifications Rxi-5 ms, 0.25 m film, 30 m long, and internal diameter 0.32 mm coated with DB-1 (J&W). The oven temperature was set at 70°C (0 min); 10°C, 150°C (5 min); 12 and 200°C (15 min); 12 and 220°C (5 min), with a hold time of 10 min for each. A temperature of 260°C was set as the inlet temperature. The flow rate was 0.6 ml/min using helium gas at a constant pressure of 90 kPa. There was an interface temperature from GC to MS, which was adjusted at a constant 280°C. The scanning range of the MS was 40–350 amu and the ionization mode (EI) was established with the mass range of 50–550 m/z. The injection volume of the sample was 1 μl. During 50 min, the total GC-MS procedure was conducted. A comparison with the National Institute of Standards and Technology (NIST) GC-MS library version 08-S was performed for the identification of compounds in the peak areas (Tareq et al., 2020).

The GC helped the blend of components to be separated into individual ones and, based on their masses, MS identified the molecules. As such, GC-MS is an analytical method combining two instruments to yield a powerful separation and identification process. From the GC-MS process, 74 compounds were isolated and 30 bioactive compounds were finally enlisted and further assessed regarding their role as ligands. Their lists as well as details of the structure and PubChem ID number are given in Table 1.

**TABLE 1:** List of tentative compounds identified in a methanol extract of *C. gigantea* leaves by GC–MS analysis.

Sl. No	Ligand name	PubChem ID	RT	Area	% PA
1	Vitamin E acetate	86472	5.484	141144	0.090494615
2	Methyl gamma-linolenate	6439889	8.260	82780	0.053074479
3	Clionasterol	457801	8.896	74665	0.047871538
4	Juniper camphor	521214	10.365	25746	0.016507073
5	Ethyl 4-fluoro-1-methyl-1H-imidazole-5-carboxylate	534521	10.986	48498	0.031094541
6	Bicyclo[3.3.1]nonane-2,4-dione, 9,9-dimethoxy-	537288	11.912	1033136	0.662396165
7	Alpha-amyrin	73170	12.567	2080749	1.334074273
8	Moretenone	604937	13.501	48457	0.031068253
9	6-Methoxy-2,5,8-trimethyl 2-(4,8,12trimethyltridecyl) 3,4-dihydrochromene	91745229	13.498	1965818	1.260386149
10	Betulinaldehyde	99615	13.498	16200780	10.38714607
11	Bicyclo[4.3.0]nonane, 1 isopropenyl-4,5-dimethyl-5-phenylsulfonylmethyl	595772	14.537	2040263	1.308116634
12	Tert-Butyl(5-isopropyl-2-methylphenoxy)dimethylsilane	13581204	14.944	2040263	1.308116634
13	Behenyl behenate	87221	15.286	764334	0.490053498
14	Ergost-5-en-3-ol, (3.beta.,24R)-	6428659	15.395	10966065	7.030903388
15	Erucic acid	5281116	15.529	18155714	11.64055393
16	Glyceryl palmitate	14900	16.255	1619813	1.038544702
17	Methyl heneicosanoate	22434	17.911	836896	0.536576696
18	Methyl palmitate	8181	20.222	5680997	3.642376828
19	Ethyl linoleate	5282184	23.559	2820957	1.808659362
20	1,3-Dihydroxypropan-2-yl (9E,12E,15E)-octadeca 9,12,15-trienoate	5367459	23.859	3420491	2.193051177
21	Palmitic acid	985	28.392	10583756	6.785785595
22	Stearic acid	5281	29.669	251160	0.161031482
23	Glyceryl monostearate	24699	31.579	19077699	12.23168553
24	Olean-12-en-3-ol, acetate, (3.beta.)-	91746489	32.070	2343815	1.502739298
25	Methyl pentadecanoate	23518	32.964	2871616	1.84113943
26	Phytol	5280435	32.955	42471	0.027230324
27	Tridecanedial	544162	32.955	10763524	6.901044026
28	Trilinolein	5322095	34.725	29317862	18.79717613
29	Methyl n-undecanoate	15607	37.086	7849076	5.032442817
30	Vitamin E	14985	46.574	2820957	1.808659362

RT, retention time; PA, peak area.

### Computational Molecular Docking Analysis

#### Protein and Ligand Preparation

Protein crystal structures are crucial for performing molecular docking simulation. In this study, we used the crystal structure of SARS-CoV-2 in concert with X77 (N-(4-tert- butylphenyl)-N-[(1R)-2-(cyclohexylamino)-2-oxo-1-(pyridin-3-yl)ethyl]-1H-imidazole-4-carboxamide) (PDB ID: 6W63) with reference to a recent study by Dubey *et al.*, who adopted the structure for performing docking studies by using some flavonoid compounds (Dubey and Dubey, 2020). The three-dimensional crystal structure of SARS-CoV-2 M^pro^ was downloaded from the Protein Data Bank in protein data bank (PDB) format having the PDB ID: 6W63 and was refined further using the protein preparation wizard of Maestro version 10.1 (Schrödinger, New York. NY, United States). Furthermore, the bond orders and charges together with the addition of hydrogen to the heavy metals; conversion of selenomethionines to the methionines; and, finally, the deletion of water were performed by using the force field OPLS_2005. Then, by setting the maximum heavy atom root mean-square-deviation to 0.30 Å, the minimization was carried out (Chowdhury et al., 2020). In addition, the structures of the chemical compounds were obtained from the PubChem database (https://pubchem.ncbi.nlm.nih.gov) and then ligands were prepared by neutralizing the pH to 7.0 ± 2.0 and minimized by the application of force field OPLS_2005 embedded in Maestro version 10.1 (Dash et al., 2015).

### Receptor Grid Generation

For prepared proteins, the receptor grids were calculated in such a way that various ligands predicted during docking were bound within the active site. In Glide, grids were brought out by keeping the default parameters of the Van der Waals scaling factor at 1.00 and the charge cutoff at 0.25, which were exposed to OPLS_2005 force field. On the centroid, a cubic box of specific dimensions was centered at the active site residues and the bounding box was set to 17 × 17 × 17 Å for docking experiments.

### Glide Standard Precision Ligand Docking

With the help of Maestro v 10.1 (Schrödinger), ligand docking was carried out, which were furthermore enforced to non-cis/trans amide bonds and the Van der Waals scaling factor; thereafter, the partial charge and cutoff values were selected (Friesner et al., 2004; Friesner et al., 2006). The ultimate scoring was executed on energy-minimized poses and represented as the glide score. For each ligand, the best-docked pose with the lowest glide score value was listed.

### Visualization of Potential Interaction

The amino acids from the receptors that bind with the ligand are examined using PDBsum (http://www.ebi.ac.uk/pdbsum), a server that provides various pictorial analyses. This server integrates LIGPLOT to show the interactions of the analyzed complexes. A LIGPLOT diagram depicts the hydrogen bonds and non-bonded interactions between each ligand molecule and the protein residues in which it interacts with each ligand molecule.

### Biological Activity Prediction

To assess the bioactivity of the lead compounds, the calculation of their activity scores as kinase inhibitors and enzyme inhibitors was completed. With the help of the software Molinspiration (www.molinspiration.com; Molinspiration, Slovensky Grob, Slovak Republic), all parameters were scrutinized properly (Rakib et al., 2020a; Rakib et al., 2020b; Tareq et al., 2020) and calculated drug-likeness scores of each compound were compared with the specific activity of each compound as well as those of standard drugs (i.e., nelfinavir and lopinavir).

### Ligand-Based ADME/T Property Analysis

The QikProp module of the Maestro version 10.1 (Schrödinger) is an easily assessable absorption, distribution, metabolism, and excretion (ADME) prediction program that has been designed specifically to obtain certain descriptors linked to ADME. It is helpful to anticipate both the physicochemical and pharmacokinetic descriptors’ relevant properties. QikProp follows Lipinski’s rule of five for the evaluation of ADME properties, ascertaining drug-similar activities of ligand molecules. ADME/T properties of the compounds (i.e., vitamin E acetate; methyl gamma-linolenate; clionasterol; juniper camphor; ethyl 4-fluoro-1-methyl-1H-imidazole-5-carboxylate; bicyclo[3.3.1]nonane-2,4-dione; 9,9-dimethoxy-; alpha.-amyrin; moretenone; 6-methoxy-2,5,8-trimethyl 2-(4,8,12trimethyltridecyl) 3,4-dihydrochromene; betulinaldehyde; bicyclo[4.3.0]nonane; 1 isopropenyl-4,5-dimethyl-5-phenylsulfonylmethyl; tert-butyl(5-isopropyl-2-methylphenoxy)dimethylsilane; behenyl behenate; ergost-5-en-3-ol, (3.beta.,24R)-; erucic acid; glyceryl palmitate; methyl heneicosanoate; methyl palmitate; ethyl linoleate; 1,3-dihydroxypropan-2-yl (9E,12E,15E)-octadeca 9,12,15-trienoate; palmitic acid; stearic acid; glyceryl monostearate; olean-12-en-3-ol; acetate, (3.beta.)-; methyl pentadecanoate; phytol, tridecanedial; trilinolein; methyl n-undecanoate; and vitamin E) were analyzed using the QikProp 3.2 module (Rahman et al., 2020).

### *In Silico* IC50 Calculation

#### Executing AutoDock

The inhibition constant in this study was determined by AutoDock 4.0 tools. AutoDock software calculates and predicts the interactions between a ligand molecule and a protein molecule, based on predefined parameters. The interactions between the molecules can be calculated at a specified user-defined region of the protein. This region must be defined by the user through the Grip map option or GridBox. Thus, the use of the GridBox at the binding site or active site, or at other essential protein regions, is essential to perform AutoDock analysis. Before executing the AutoDock, the “.pdb” files of the protein and ligand have to be moved into one folder.

Analysis in AutoDock can be divided into the following categories: a) Initialising molecules; and b) Running AutoGrid.

#### Initialising Molecules

Initialising the molecule involved the addition of hydrogen atoms. The ligand molecule required the addition of the Gasteiger charge, the identification of aromatic carbons, the detection of rotatable bonds, and the setting of the torsional degrees of freedom (TORSDOF) value. The protein must be initialised manually, whereas the ligand is automatically initialised when opened in the tool. The receptor and ligand files were saved in.pdbqt format. Next, the receptor was opened again using the “Grid” menu and the “Macromolecules” sub-menu, and “open” was selected. The ligand opens using the “Set Map Types” sub-menu and “Open Ligand.” Then, the GridBox is set in AutoDock to cover the identified binding sites. AutoDock only analyses the ligand molecule’s interactions and the amino acids that are present within the GridBox. The GridBox’s size can be increased or decreased using the number of points in the X/Y/Z dimension. The position of the GridBox can be adjusted to cover the binding site or binding residues, using the “Center GridBox” field that moves the GridBox in the X, Y, and Z-axis.

#### Running AutoGrid and AutoDock

After setting up the GridBox, the AutoGrid file was saved. When in the “Grid” menu, select the “Output” and “Save GPF” sub-menu. The file saves in.gpf format. Meanwhile, the AutoDock file saving procedure is to choose the “Docking” menu and select “Lamarckian GA” in the “Output” menu. The file saves in.dpf format. The AutoGrid and AutoDock programs was run through a command prompt directed to the folder to be docked using the command: *autogrid4 -p control.gpf -l control.glg*. After the AutoGrid calculation was completed successfully, AutoDock was run using the command: *autodock4 -p control.dpf -l control.dlg*. The results of the docking calculations are obtained in a notepad file format, reporting the values for binding energy and the estimated inhibition constant.

## Results

### GC-MS Analysis

**FIGURE 1:**
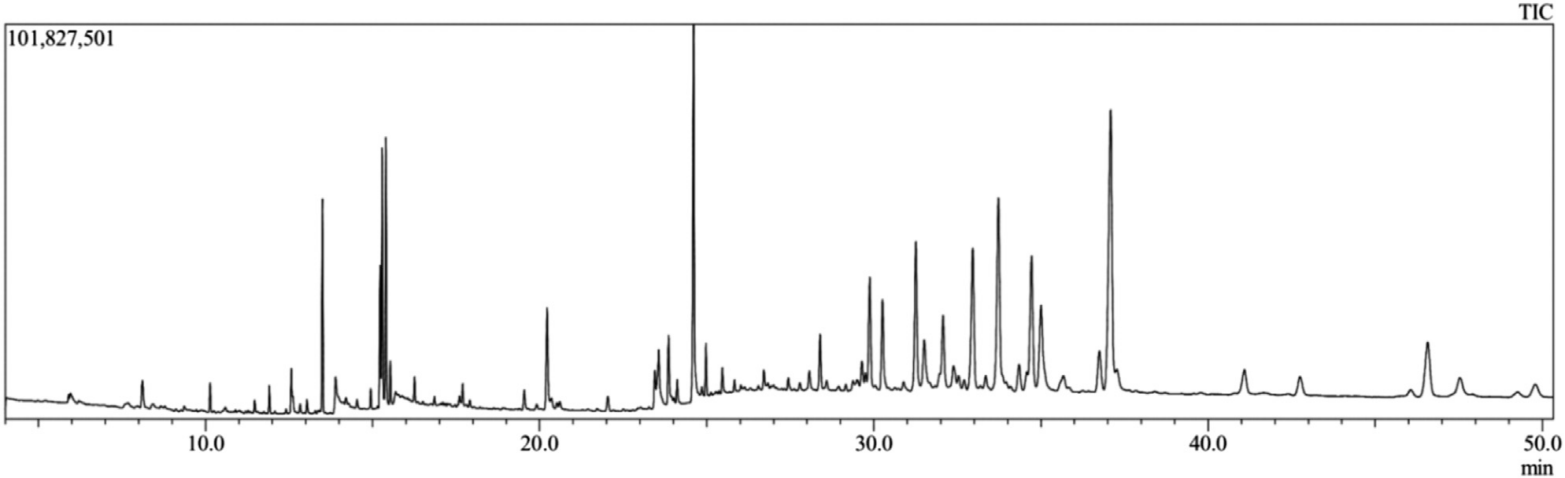
Total ionic chromatogram (TIC) of methanol extract of *C. gigantea* leaves by GC-MS.

From the methanolic leaf extract of *C. gigantea*, 52 compounds were eluted in between the retention time of 5 and 32 min (Figure 1). Thirty bioactive compounds were selected for this study (Table 1). The compound identification was carried out in comparison with the NIST GC-MS library version 08-S. The major phytochemicals included phenols, sterols, terpenoids, esters, and other organic compounds. The major compounds identified are trilinolein (18.79%), glyceryl monostearate (12.23%), erucic acid (11.64%), betulinaldehyde (10.38%), ergost-5-en-3-ol, (3.beta.,24R)- (7.03%), tridecanedial (6.90%), palmitic acid (6.78%), and methyl n-undecanoate (5.03%).

### Molecular Docking

A virtual screening process, including molecular docking, is a feasible approach for identifying antiviral therapeutics among diverse peptide sequences available in the online-based database. Notably, the interaction type, and the binding energy in conjunction with the bond distance of interaction between a protein substrate and a ligand, can be evaluated through the utility of molecular docking. Therefore, screening a ligand among numerous plant sources based on binding energy assessment is possible within minimum time by running molecular docking to evaluate the best ligand candidates according to the docking interaction.

### Docking Interaction

**FIGURE 2:**
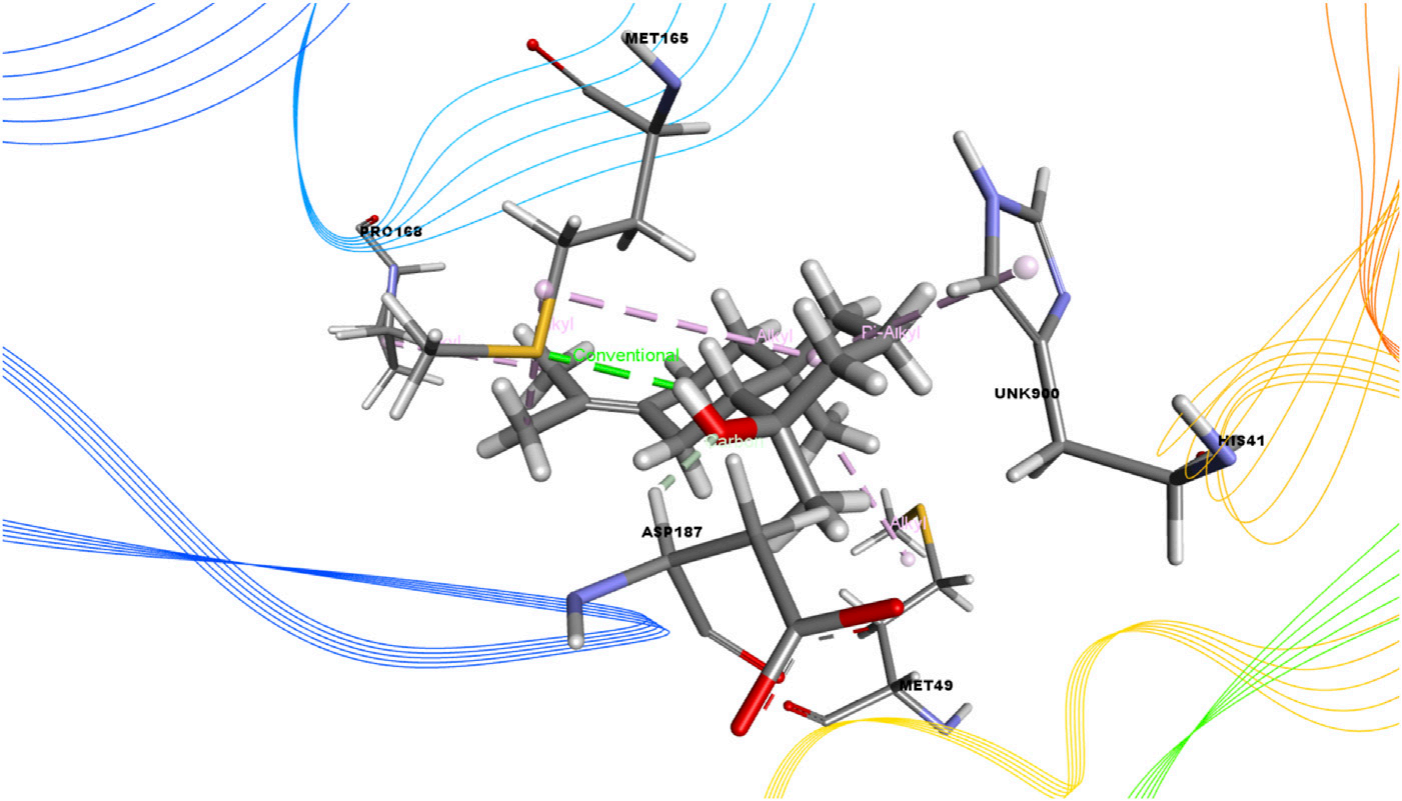
3D interactions of Juniper camphor with the active site of SARS-CoV-2 main protease (PDB ID: 6W63).

In this study, we determined the interaction between several isolated compounds of *C. gigantea* and the M^pro^ enzyme. The docking results of the compounds that docked with the M^pro^ enzyme of SARS-CoV-2 are presented in Tables 2, 3; Figures 2 through 5. Meanwhile, the two- and three-dimensional interactions of nelfinavir and lopinavir with the active site of the SARS-CoV-2 M^pro^ are shown in Figures 6 and 7.

**TABLE 2:** Molecular docking study of major bioactive compounds from methanol extract of C. gigantea.

S/L No	Compound name	Docking score
1.	Vitamin E acetate	−2.846
2.	Juniper camphor	−**6.06**
3.	Ethyl 4-fluoro-1-methyl-1H-imidazole-5-carboxylate	−**5.558**
4.	Alpha-amyrin	−5.366
5.	6-Methoxy-2,5,8-trimethyl 2-(4,8,12trimethyltridecyl) 3,4-dihydrochromene	−2.506
6.	Betulinaldehyde	−5.195
7.	Bicyclo[4.3.0]nonane, 1 isopropenyl-4,5-dimethyl-5-phenylsulfonylmethyl	−**5.808**
8.	Glyceryl palmitate	−1.141
9.	Methyl palmitate	0.433
10.	Palmitic acid	1.479
11.	Stearic acid	1.039
12.	Olean-12-en-3-ol, acetate, (3.beta.)-	−**5.588**
13.	Methyl pentadecanoate	0.806
14.	Tridecanedial	0.557
15.	Methyl n-undecanoate	1.689
16.	Vitamin E	−4.295

Bold text indicates the best docking scores.

**TABLE 3:** Ligand interactions with amino acids of the protein active site.

S/L no	Compound name	Hydrogen bond interaction	Hydrophobic bond interaction
1.	Vitamin E acetate	—	HIS41 (Pi-Alkyl)
			HIS41 (Pi-Alkyl)
			TYR 54 (Pi-Alkyl)
			CYS 44 (Alkyl)
			MET 49 (Alkyl)
			Met 165 (Alkyl)
			PRO 168 (Alkyl)
			PRO 168 (Alkyl)
2.	Juniper camphor	MET 165	MET 49 (Alkyl)
			MET 165 (Alkyl)
			PRO 168 (Alkyl)
			HIS 41 (Pi-Alkyl)
			ASP 147 (Carbon)
3.	Ethyl 4-fluoro-1-methyl-1H-imidazole-5-carboxylate	CYS 44	HIS 41 (Carbon)
			ASP 187 (Fluorine)
			MET 165 (Pi-Sulfur)
			MET 49 (Alkyl)
			HIS 41 (Pi-Pi-T-Shaped)
			ARG (Amide-Pi-Stacked)
4.	Alpha-amyrin	—	HIS 41 (Pi-Alkyl)
			HIS 41 (Pi-Alkyl)
			HIS 41 (Pi-Alkyl)
			HIS 41 (Pi-Alkyl)
			CYS 141 (Alkyl)
			CYS 141 (Alkyl)
			CYS 44 (Alkyl)
			MET 165 (Alkyl)
			MET 49 (Alkyl)
			MET 49 (Alkyl)
			MET 49 (Alkyl)
5.	6-Methoxy-2,5,8-trimethyl 2-(4,8,12trimethyltridecyl) 3,4-dihydrochromene	—	CYS 145 (Pi-Alkyl)
			CYS 145 (Pi-Alkyl)
			HIS 41 (Pi-Alkyl)
			HIS 41 (Pi-Alkyl)
			THR 24 (Carbon)
6.	Betulinaldehyde	GLN 189	CYS 145 (Alkyl)
			MET 165 (Alkyl)
			MET 49 (Alkyl)
			MET 49 (Alkyl)
			MET 49 (Alkyl)
			CYS 41 (Alkyl)
			HIS 41 (Pi-Alkyl)
7.	Bicyclo[4.3.0]nonane, 1 isopropenyl-4,5-dimethyl-5-phenylsulfonylmethyl	CYS 145	MET 49 (Alkyl)
			MET 49 (Alkyl)
			MET 165 (Alkyl)
			MET 165 (Alkyl)
			CYS 44 (Alkyl)
			CYS 44 (Alkyl)
			HIS 41 (Pi-Alkyl)
			HIS 41 (Pi-Alkyl)
			HIS 41 (Pi-Alkyl)
			HIS 41 (Pi-Alkyl)
8.	Glyceryl palmitate		MET 43 (Alkyl)
9.	Methyl palmitate	SER 46	MET 165 (Alkyl)
10.	Palmitic acid	SER 46	MET 165 (Alkyl)
			HIS 41 (Alkyl)
			THR 145 (Carbon)
11.	Stearic acid	GLN 189	CYS 44 (Alkyl)
		GLN 192	MET 49 (Alkyl)
		THR 190	GLN 189 (Carbon)
		THR 190	THR 190 (Acceptor-Acceptor)
12.	Olean-12-en-3-ol, acetate, (3.beta.)-	THR 26	MET 165 (Alkyl)
			MET 165 (Alkyl)
			MET 165 (Alkyl)
			HIS 41 (Pi-Alkyl)
			HIS 41 (Pi-Alkyl)
			HIS 41 (Pi-Alkyl)
			HIS 41 (Pi-Sigma)
			MET 49 (Alkyl)
			MET 49 (Alkyl)
			THR 25 (Carbon)
			THR 26 (Carbon)
13.	Methyl pentadecanoate	—	MET 49 (Alkyl)
			MET 165 (Alkyl)
			LEU 167 (Alkyl)
			PRO 168 (Alkyl)
			CYS 44 (Alkyl)
			CYS 44 (Carbon)
			CYS 44 (Carbon)
14.	Tridecanedial	CYS 44	THR 190 (Acceptor-Acceptor)
		GLN 192	
15.	Methyl n-undecanoate		HIS 41 (Pi-Alkyl)
			PRO 168 (Carbon)
16.	Vitamin E	—	CYS 44 (Alkyl)
			MET 49 (Alkyl)
			MET 49 (Alkyl)
			MET 165 (Alkyl)
			MET 165 (Alkyl)
			LEU 167 (Alkyl)
			PRO 168 (Alkyl)
			PRO 168 (Alkyl)
Standard			
17.	Nelfinavir	HIS 41	CYS 44 (Pi-Sulfur)
		GLU 166	CYS 145 (Pi-Sulfur)
			MET 49 (Pi-Sulfur)
			MET 165 (Amide-Pi-Stacked)
			HIS 41 (Pi-Alkyl)
			GLU 166 (Carbon)
18.	Lopinavir	GLN 189	PRO 168 (Pi-Sigma)
		GLU 166	PRO 168 (Alkyl)
		CYS 141	HIS 41 (Pi-Alkyl)
		THR 26	HIS 41 (Pi-Alkyl)
			HIS 41 (Pi-Pi-T Shaped)
			GLN 189 (Amide-Pi-Stacked)
			MET 165 (Pi-Sulfur)
			MET 49 (Pi-Sulfur)
			CYS 145 (Pi-Sulfur)

**FIGURE 3:**
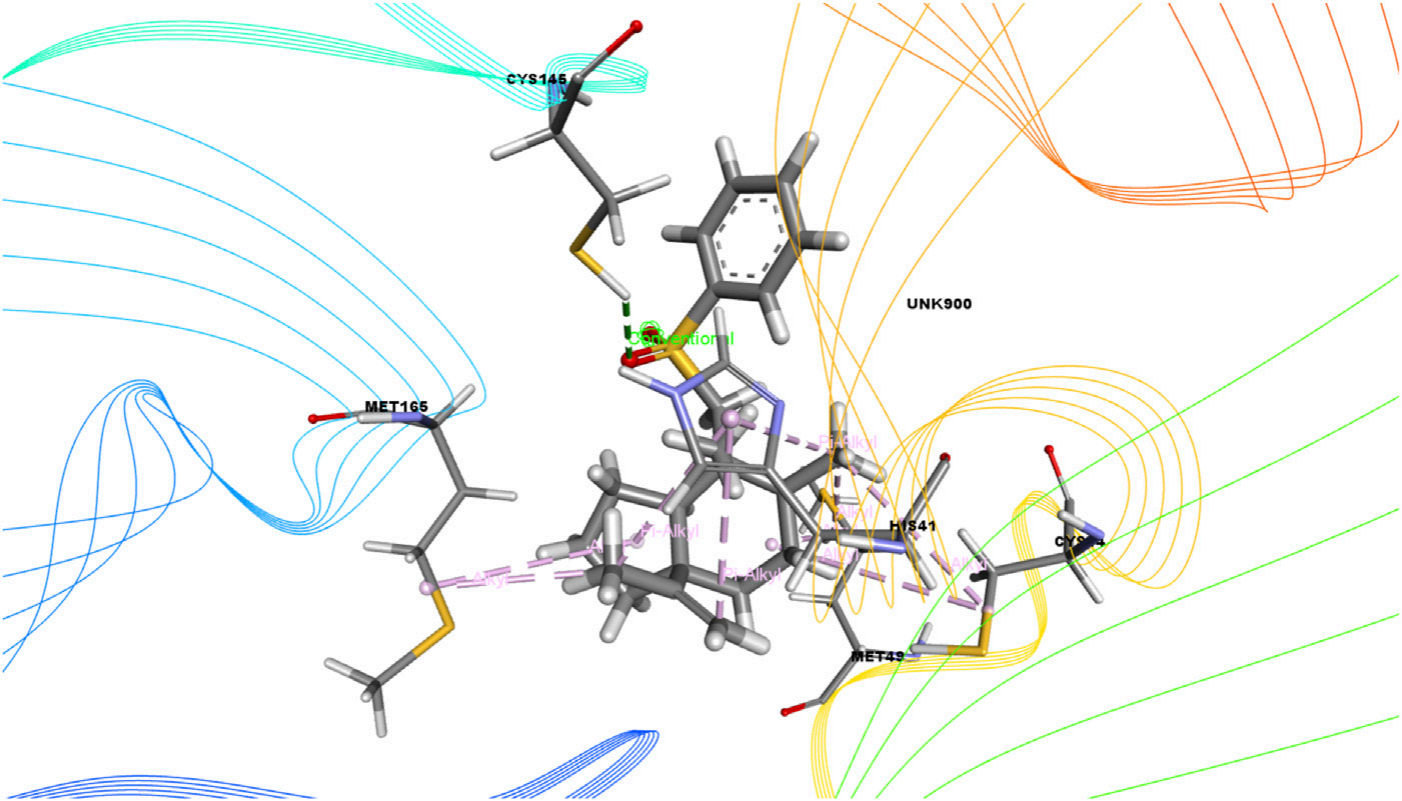
3D interactions of Bicyclo[4.3.0]nonane, 1 isopropenyl-4,5-dimethyl-5-phenylsulfonylmethyl with the active site of SARS-CoV-2 main protease (PDB ID: 6W63).

**FIGURE 4:**
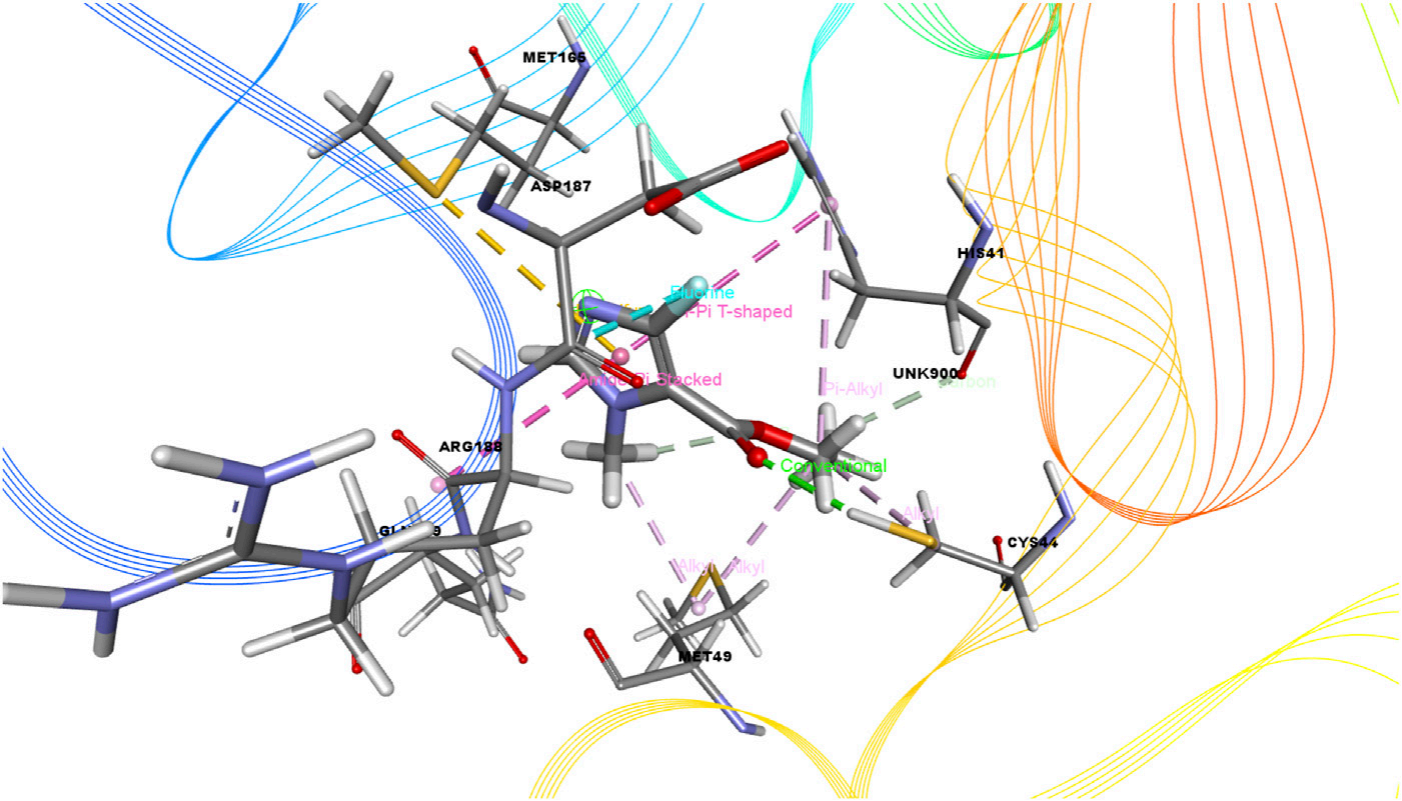
3D interactions of Ethyl 4-fluoro-1-methyl-1H-imidazole-5-carboxylate with the active site of SARS-CoV-2 main protease (PDB ID: 6W63).

**FIGURE 5:**
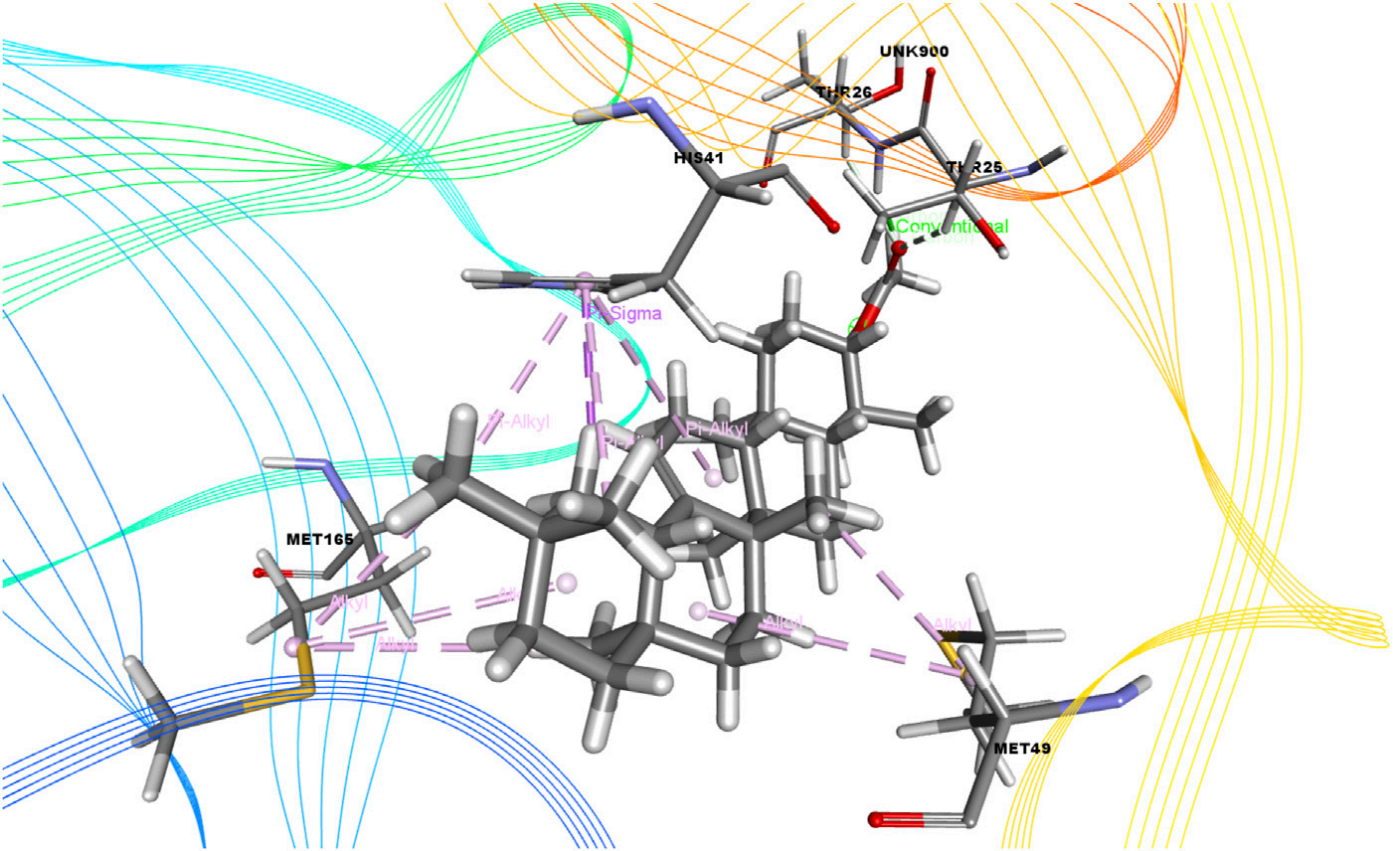
3D interactions of Olean-12-en-3-ol, acetate, (3.beta.)- with the active site of SARS-CoV-2 main protease (PDB ID: 6W63).

**FIGURE 6:**
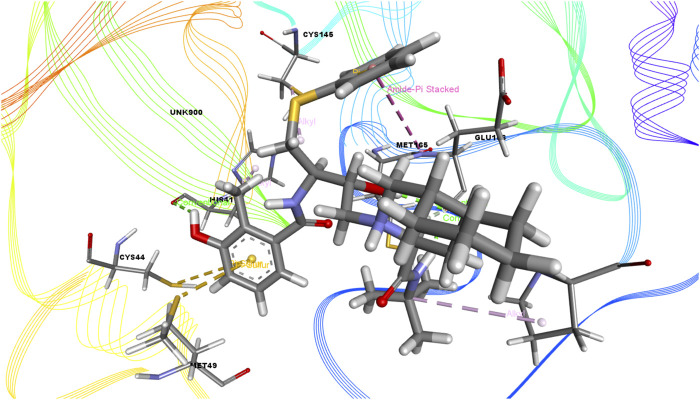
3D interactions of Nelfinavir with the active site of SARS-CoV-2 main protease (PDB ID: 6W63).

**FIGURE 7:**
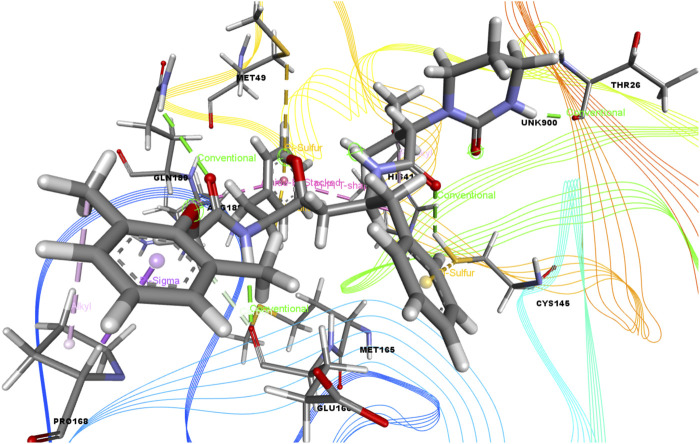
3D interactions of Lopinavir with the active site of SARS-CoV-2 main protease (PDB ID: 6W63).

Among the molecular docking results, nelfinavir and lopinavir were confirmed to have docked with the main protease and had docking scores of −7.596 and −8.251 kcal/mol, respectively. According to the docking scores, juniper camphor was better in comparison with the standard drugs. Bicyclo[4.3.0]nonane, 1 isopropenyl-4,5-dimethyl-5-phenylsulfonylmethyl, olean-12-en-3-ol, acetate, (3.beta.)-, and ethyl 4-fluoro-1-methyl-1H-imidazole-5-carboxylate also exhibited better scores in a moderate fashion. Moreover, methyl pentadecanoate shows lowest docking score (0.806 kcal/mol). If arranging these compounds according to their scores, then the arrangement would be, from highest to lowest, juniper camphor (−6.06 kcal/mol); bicyclo[4.3.0]nonane, 1 isopropenyl-4,5-dimethyl-5-phenylsulfonylmethyl (−5.808 kcal/mol); olean-12-en-3-ol, acetate, (3.beta.)- (−5.588 kcal/mol); and ethyl 4-fluoro-1-methyl-1H-imidazole-5-carboxylate (−5.558 kcal/mol).

Again, the antiviral drug nelfinavir demonstrated interactions with HIS 41 and GLU 166 (two interactions) and three H-bonds with CYS 44, CYS 145, and MET 49 together with three pi-sulfur bonds together with one amide-pi-stacked, pi-alkyl, and carbon bond each with MET 165, HIS 41, and GLU 166, respectively. Lopinavir interacted with the protein through four H-bonds with GLN 189, GLU 166, CYS 141, and THR 26; nine hydrophobic bonds and three pi-sulfur bonds with MET 165, MET 49, and CYS 145; two pi-alkyl and pi-pi-T shaped bonds with HIS 41; one pi-sigma bond and one alkyl bond with PRO 168; and an amide-pi-stacked bond with GLN 189. Moreover, juniper camphor displayed five hydrophobic bonds, where three were alkyl bonds with MET 49, MET 165, and PRO 168; one was a pi-alkyl bond with HIS 41; and one was a carbon bond with ASP 147. These compounds also formed H-bonds with MET 165.

On the other hand, bicyclo[4.3.0]nonane, 1 isopropenyl-4,5-dimethyl-5-phenylsulfonylmethyl formed one H-bond with CYS 145; six alkyl bonds with MET 49, MET 49, MET 165, MET 165, CYS 44, and CYS 44; and four pi-alkyl bonds with HIS 41. Besides, olean-12-en-3-ol, acetate, (3.beta.)- showed a single H-bond, five alkyl bonds, three pi-alkyl bonds, two carbon bonds, and a single pi-sigma bond with THR 26, MET 165, MET 165, MET 165, MET 49, MET 49, HIS 41, HIS 41, HIS 4, HIS 41, THR 25, and THR 26, respectively.

As seen in Table 3, we also found that ethyl 4-fluoro-1-methyl-1H-imidazole-5-carboxylate interacted with the protein through a single H-bond and six hydrophobic bonds as follows: HIS 41 (carbon), ASP 187 (fluorine), MET 165 (pi-sulfur), MET 49 (alkyl), HIS 41 (pi-pi-T-shaped), and ARG (amide-pi-stacked).

### Ligand-Based Biological Activity Parameter Prediction

Specific parameters, protease inhibitors, and enzyme inhibitors of all selected compounds are shown in Table 4.

**TABLE 4:** Biological activity parameter of the selected bioactive compounds.

S/L No.	Compound name	Biological activity prediction	
		Protease inhibitor	Enzyme inhibitor
1.	Vitamin E acetate	0.22	0.16
2.	Methyl gamma-linolenate	0.03	0.23
3.	Clionasterol	0.07	0.51
4.	Juniper camphor	−0.70	0.29
5.	Ethyl 4-fluoro-1-methyl-1H-imidazole-5-carboxylate	−0.98	−0.12
6.	Bicyclo[3.3.1]nonane-2,4-dione, 9,9-dimethoxy-	−0.67	−0.12
7.	alpha.-Amyrin	0.19	0.60
8.	Moretenone	0.00	0.37
9.	6-Methoxy-2,5,8-trimethyl 2-(4,8,12trimethyltridecyl) 3,4-dihydrochromene	0.19	0.13
10.	Betulinaldehyde	0.20	0.53
11.	Bicyclo[4.3.0]nonane, 1 isopropenyl-4,5-dimethyl-5-phenylsulfonylmethyl	0.30	0.27
12.	Tert-Butyl(5-isopropyl-2-methylphenoxy)dimethylsilane	0.53	1.03
13.	Behenyl behenate	0.45	−0.06
14.	Ergost-5-en-3-ol, (3.beta.,24R)-	0.01	0.50
15.	Erucic acid	0.18	0.23
16.	Glyceryl palmitate	0.13	0.24
17.	Methyl heneicosanoate	0.06	0.04
18.	Methyl palmitate	−0.13	0.04
19.	Ethyl linoleate	0.03	0.18
20.	1,3-Dihydroxypropan-2-yl (9E,12E,15E)-octadeca 9,12,15-trienoate	0.20	0.43
21.	Palmitic acid	−0.04	0.18
22.	Stearic acid	0.06	0.20
23.	Glyceryl monostearate	0.15	0.22
24.	Olean-12-en-3-ol, acetate, (3.beta.)-	0.06	0.48
25.	Methyl pentadecanoate	−0.20	0.01
26.	Phytol	0.00	0.31
27.	Tridecanedial	−0.17	0.11
28.	Trilinolein	−2.33	−3.17
29.	Methyl n-undecanoate	−0.56	−0.17
30.	Vitamin E	0.28	0.24
Standard drugs			
31.	Nelfinavir	0.58	−0.02
32.	Lopinavir	0.42	−0.37

### Ligand-Based ADME/T Prediction

Pharmacokinetic and physicochemical properties of the 30 selected bioactive compounds were screened for drug-candidacy using the QikProp module of the Schrödinger suite-Maestro version 10.1. The ligand molecule’s similar drug attitude was determined using ADME properties by the QikProp module of Schrodinger Maestro v10.1. The ADME/T properties of the selected compounds were determined with the QikProp module of Schrodinger, shown in Table 5.

**TABLE 5:** ADME and drug likeness properties of selected bioactive isolated compounds from methanol extract of *C. gigantea*.

Compound name	MW^a^	HB donors^b^	HB acceptors^c^	SASA^d^	QPlogPo/w^e^	QPlogBB^f^	QPlogS^g^	%Human oral absorption^h^
Vitamin E acetate	472.75	0	3.25	772.266	8.151	−0.358	−7.034	100
Methyl gamma-linolenate	292.461	0	2	609.328	5.485	−0.605	−4.60	100
Clionasterol	414.713	1	1.7	756.851	7.473	−0.337	−8.353	100
Juniper camphor	222.37	1	0.75	450.587	3.96	0.391	−3.965	100
Ethyl 4-fluoro-1-methyl-1H-imidazole-5-carboxylate	172.159	0	3.5	390.235	1.207	−0.098	−1.605	92.876
Bicyclo[3.3.1]nonane-2,4-dione, 9,9-dimethoxy-	212.245	0	5.5	407.383	0.545	−0.235	−0.732	86.661
Alpha-amyrin	426.724	1	1.7	674.998	6.947	0.191	−7.806	100
Moretenone	424.709	0	2	692.442	7.036	0.163	−8.129	100
6-Methoxy-2,5,8-trimethyl 2-(4,8,12trimethyltridecyl) 3,4-dihydrochromene	430.713	0	1.5	753.331	6.351	0.986	−12.387	100
Betulinaldehyde	440.708	1	3.7	690.396	5.918	−0.34	−7.009	100
Bicyclo[4.3.0]nonane, 1 isopropenyl-4,5-dimethyl-5-phenylsulfonylmethyl	346.527	0	4	588.762	4.457	−0.013	−4.629	100
Tert-Butyl(5-isopropyl-2-methylphenoxy)dimethylsilane	264.482	0	0.85	550.079	4.574	0.746	−7.589	100
Behenyl behenate	649.178	0	3	1729.5	15.728	−0.045	−27.42	100
Ergost-5-en-3-ol, (3.beta.,24R)-	400.687	1	1.7	745.016	7.225	−0.283	−8.295	100
Erucic acid	338.573	1	2.75	861.367	6.08	−0.755	−10.855	100
Glyceryl palmitate	330.507	2	5	886.624	4.614	−1.036	−10.395	100
Methyl heneicosanoate	340.588	1	3	903.182	6.741	0.158	−11.692	100
Methyl palmitate	270.454	0	2	721.861	5.815	−0.926	−6.57	100
Ethyl linoleate	308.503	0	3	670.733	4.826	0.25	−7.357	100
1,3-Dihydroxypropan-2-yl (9E,12E,15E)-octadeca 9,12,15-trienoate	352.513	2	4	812.017	4.485	−0.584	−9.404	100
Palmitic acid	256.428	1	2	678.44	5.303	−1.492	−5.64	87.371
Stearic acid	284.481	1	2.75	770.357	4.965	−0.704	−9.168	100
Glyceryl monostearate	358.56	2	5	960.581	5.385	−1.107	−11.735	100
Olean-12-en-3-ol, acetate, (3.beta.)-	454.735	0	2	725.863	7.292	−0.223	−8.762	100
Methyl pentadecanoate	256.428	0	2	688.711	5.42	−0.848	−6.105	100
Phytol	296.535	1	1.7	639.152	5.819	−0.767	−5.024	100
Tridecanedial	212.331	0	4	573.453	2.492	−1.633	−3.036	88.392
Trilinolein	—	—	—	—	—	—	—	—
Methyl n-undecanoate	200.32	0	2	556.54	3.834	−0.542	−4.251	100
Vitamin E	430.713	1	1.5	732.405	7.983	−0.522	−6.998	100
Nelfinavir	567.785	4	9.95	922.327	4.344	−0.966	−5.652	79.965
Lopinavir	628.81	9.833	3.083	822.525	4.933	0.195	−7.416	96.153

a Molecular weight (acceptable range: < 500).

b Hydrogen bond donor (acceptable range: ≤5).

c Hydrogen bond acceptor (acceptable range: ≤10).

d Total Solvent Accessible Surface Area in using a probe with a 1.4 radius (acceptable range: 300–1,000).

e Predicted octanol/water partition coefficient (acceptable range: − 2–6.5).

f Predicted blood-brain partition co-efficient (acceptable range: −3–1.2).

g Predicted aqueous solubility, S in mol/dm^−3^ (acceptable range: −6.5–0.5).

h Predicted human oral absorption on 0–100% scale (<25% is poor and >80% is high).

### Visualisation of Interactions

The docking conformation of the ligands and receptors were determined by selecting the pose with the highest affinity (most negative Gibbs free energy). The results from each affinity value of the conformation were obtained in the notepad file. The visualization of three-dimensional (3D) and two-dimensional (2D) structures from the docking calculations were carried out. The visualization and potential interaction between each ligand molecule with protein residues results are presented in Supplementary Table S1; Supplementary Figure S1. The interaction of the receptor’s amino acid with the ligand in 3D and 2D were saved as image files.

### *In Silico* Inhibition Constant Calculation

The current study determined the inhibition constant of major bioactive compounds from methanol extract of *C. gigantea*. toward the modeled SARS-CoV-2 M^pro^. The theoretical half-maximal inhibitory concentration (IC_50_) was calculated using AutoDock 4.0 tools, and the results of the analysis are shown in Table 6.

**TABLE 6:** Inhibition constant (IC_50_) of major bioactive compounds from methanol extract of *C. gigantea.*

Compound name	Inhibition constant (IC_50_) (µM)
Juniper camphor	281.23
Ethyl 4-fluoro-1-methyl-1H-imidazole-5-carboxylate	313.40
Bicyclo[4.3.0]nonane, 1 isopropenyl-4,5-dimethyl-5-phenylsulfonylmethyl	727.23
Olean-12-en-3-ol, acetate, (3.beta.)-	845.30

## Discussion

Currently, SARS-CoV-2 infection has emerged as a global pandemic and requires an international public health emergence (Matveeva et al., 2020; Remali and Aizat, 2021). However, different research organizations and pharmaceutical companies are working relentlessly for the management of the infection. By far, several vaccines have been made available as immunomodulation is characterized as one of the pivotal characteristics in the maintenance of human health and productivity (Tiwari et al., 2018; Adhikari et al., 2020; Tahir Ul Qamar et al., 2020; Tallei et al., 2020 ).

Researchers are learning new things about this virus every day about the mutation of the virus (Sinha et al., 2021). Surprisingly, according to Centers for Disease Control and Prevention (CDC), multiple SARS-CoV-2 variants are available globally. In United Kingdom, the variant of SARS-CoV-2 showed a large number of mutations In South Africa, another variant emerged independently and share several characteristics with other strain. As the mutation of the virus is rising rapidly, it is crucial to identify potential therapeutic agents to overcome the pathetic situation. So far, we know that initially, some patients having attacked by COVID-19 may not figure out any symptoms, and hence they may carry the virus for two days or up to two weeks without noticing any symptoms (Tahmasbi et al., 2021). Some common symptoms have explicitly been notified to COVID-19. These include shortness of breath, a low-grade fever that gradually increases in temperature, having a cough that gets more severe over time, and fatigue. Less common symptoms include chills, repeated shaking with colds, sore throat, headache, muscle aches and pains, loss of taste, and smell loss. Some people may need to seek emergency medical services to have any of the symptoms more severe, like trouble breathing with persistent pain or pressure in the chest and excessive drowsiness (——). Although several research works are being carried out in renowned laboratories in various countries, the vaccine development for a disease generally requires a long time. Despite plenty of experimental trials related to COVID-19 treatment and medications, scientists are still seeking specific therapeutic drugs. Several previous studies have already performed for the identification of potential therapeutic agents against SARS-CoV-2 from medicinal plants (Rabaan et al., 2020; Shoaib et al., 2020; Emirik, 2020). Medicinal plants are abundant with plenty of phytocompounds and have been used to treat several kinds of diseases (Shree et al., 2020). Different secondary metabolites, including lignin’s, glycosides, alkaloids, terpenoids, and amino acids that are fundamental for the functioning and growth of plants as well as having numerous pharmacological attributes to fight against several abnormal conditions (Rahman et al., 2013; Emran et al., 2018; Diniz et al., 2021 ). We had selected the plant *C. gigantea* based on their traditional use and ethnomedicinal value and their contribution as an antiviral drug for certain viruses that were analyzed before. For this study, with the help of the GC-MS technique, seventy-four compounds had been isolated, among which thirty bioactive compounds were enlisted and subjected to play their role as ligands. In recent times, researchers are working to find out some potential lead compounds from medicinal plants that are active against several enzymes and other proteins responsible for viral replication and growth (Gupta et al., 2020b; Krupanidhi et al., 2020; Pendyala and Patras, 2020 ). Several studies reported the inhibitory potential of several herb derived phytochemicals against SARS-CoV-2 (Dhama et al., 2018; Gupta et al., 2020b; Jalali et al., 2020).

The advancement of numerous computational technology has affected vastly in biology and computational biology, incorporating both computational algorithms and biological data, which has emerged as a breakthrough in modern drug discovery. High-throughput screening (HTS) often requires the screening of the plenty of chemical compounds specific for a targeted receptor, utilizes molecular docking as essential tools (Brooijmans and Kuntz, 2013). We predicted the interaction between the SARS-CoV-2 M^pro^ and the targeted phytochemical through molecular docking simulation in the current study. The molecular docking analysis findings revealed that the selected phytocompounds were able to interact with the binding pockets of the SARS-CoV-2 M^pro^. The compound Juniper camphor exhibited the most significant interaction compared with other compounds. Like two standard drugs, nelfinavir, and lopinavir, it yielded hydrogen bonding with Met165 residue. Besides, our selected compounds were able to interact with the SARS-CoV-2 M^pro^, and previous other studies also suggested the residues were responsible for interacting with SARS-CoV-2 M^pro^ (Joshi et al., 2020; Khan et al., 2020; Wu et al., 2020).

Molinspiration is used to predict the biological activity parameters like protease inhibitory activity and enzyme inhibitory action. The increase scores specify better inhibition properties (Yamamoto et al., 2004; Chang et al., 2019; Das et al., 2020; Gentile et al., 2020; Li et al., 2020); most of the targeted compounds were shown greater inhibition properties.

QikProp module of the Schrödinger suite predicts ADMET properties related to molecular weight, partition coefficient (QPlogPo/w), stars, % oral absorption values, and Lipinski’s properties (Rule of five). Different ADME parameters were predicted, as the molecular weight ranged between 200.32 and 649.178, where only one phytochemical was outside the specified range. The estimated number of hydrogen bond donors (HB donors) by the solute to water molecules in an aqueous solution lay in the range between 1 and 2. In contrast, in the case of hydrogen bond acceptor (HB acceptors), the number was found between 0.75 and 5.5. The value of total solvent accessible surface area (SASA) ranged from 390.235 to 1729.5, where only Behenyl behenate was recorded outside the acceptable range. The value of the octanol/water partition coefficient (QPlog Po/w) lay in the range between 0.545 and 15.728. All the bioactive compounds have shown good Brain/blood partition coefficient (QPlogBB), the drug’s ability to pass through the blood-brain barrier (BBB). The QPlogBB value ranged from −1.633 to 0.986 suggested all the compounds are within the specified limits; aqueous solubility, log S (QPlogS) ranged between −27.42 and −0.732. Interestingly, all the compounds except Behenyl behenate fulfilled the Lipinski’s ROF as it was found that Lipinski violations were ≤1. The percent of human oral absorption was high for all the selected compounds. All the phytochemicals exceeded 80% and showed more oral absorption than both the standard drugs. Notably, some phytochemicals exhibited 100% oral bioavailability, which enhanced the drug candidacy of those compounds.

Enzyme activation or inhibition is the result of the binding interaction between a small molecule ligand and an enzyme protein. The protein is supposed to be a receptor, and thus binding with the ligand may result in agonism or antagonism. As a form of bioinformatics analysis, Molecular docking simulation reveals the binding affinities of ligand molecules with a specific receptor, where the lower binding energy predicts the greater binding affinity (Brooijmans and Kuntz, 2003; Lalitha and Sivakamasundari, 2010; Ahmed et al., 2019; Rakib et al., 2020c; Rakib et al., 2020d; Jahan et al., 2020; Dutta et al., 2019). Despite showing lower binding affinities than nelfinavir and lopinavir, our selected compounds interacted with the active pockets of the SARS-CoV-2 M^pro^ enzyme like the standard compounds. Among the selected 30 ligands, 16 ligands docked with active pockets of SARS-CoV-2 M^pro^ enzymes. Four of them, namely Juniper camphor, Ethyl 4-fluoro-1-methyl-1H-imidazole-5-carboxylate, Bicyclo[4.3.0]nonane, 1 isopropenyl-4,5-dimethyl-5-phenylsulfonylmethyl and Olean-12-en-3-ol, acetate, (3.beta.) possessed docking scores more likely to nelfinavir. Moreover, those compounds also followed the Lipinski’s rule of five for drug-likeness properties. Hence, these four may be potential inhibitors of COVID-19, which warrants further investigation.

While so many synthetic bioactive compounds are undergoing clinical trials, including medicinal plants derived phytocompounds, this purely bioinformatic work was limited by a lack of direct experimental evidence. We were unable to conduct follow-up experiments using other techniques, such as Western blotting or qRT-PCR, to confirm our bioactive compounds’ antiviral properties generated only from computational prediction. This study could be expanded to examine the antiviral potentiality of plant-derived bioactive compounds, specifically from several traditional medicinal plants available in the Bangladesh region. Based on results from this study, the top compounds that might turn out to be prospective anti–SARS-CoV-2 lead molecules warrant further investigation and possible application in drug development processes against COVID-19.

## Conclusion

Increasing use of HTS and molecular docking simulation is emerged as a new era in the field of drug discovery. Medicinal plant derived phytochemicals are used for the treatment from since the dawn of civilization. Recently, the enriched extraction procedures and sophisticated computer-aided program have already added a new dimension in the field of drug discovery. To sum up, this study provides significant data for the use of isolated phytocompounds from *C. gigantea* for the treatment of COVID-19 by targeting one of the crucial enzymes that is essential replication of SARS-CoV-2. Further experimental analysis might be needed to justify the activity of the phytochemicals at the molecular level. In addition, many more such bioactive ingredients from medicinal plants existing in the rich Bangladeshi biodiversity need to be further explored. The study initiated the window toward plant-based therapy against COVID-19, though extensive research and wet lab validation need to make it usable for the patient.

## Data Availability

The datasets presented in this study can be found in online repositories. The names of the repository/repositories and accession number(s) can be found in the article/Supplementary Material.
