# Supplementary material for: Appraisals of the Bangladeshi Medicinal Plant Calotropis gigantea Used by Folk Medicine Practitioners in the Management of COVID-19: A Biochemical and Computational Approach
Source: Front Mol Biosci. 2021 May 26;8:625391. doi: 10.3389/fmolb.2021.625391 (PMC8187851; doi:10.3389/fmolb.2021.625391)
Supplement: Supplementary file 1 [file Table1.docx]

Supplementary Materials

# Supplementary Figures and Tables

## Supplementary Figures

| 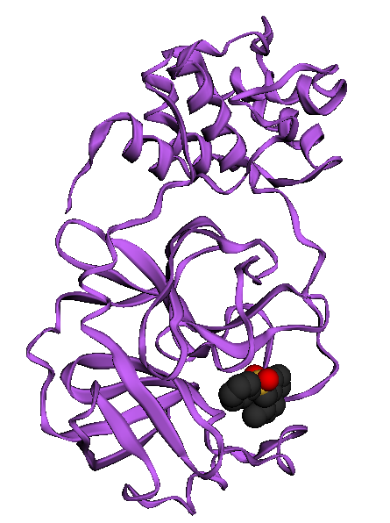 | 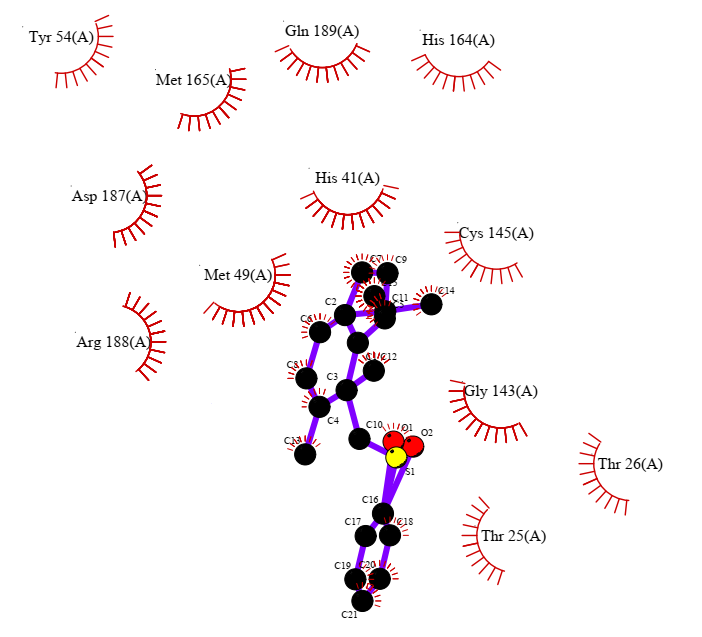 |
| --- | --- |
| **Bicyclo[4.3.0]nonane, 1 isopropenyl-4,5-dimethyl-5-phenylsulfonylmethyl** | |

| 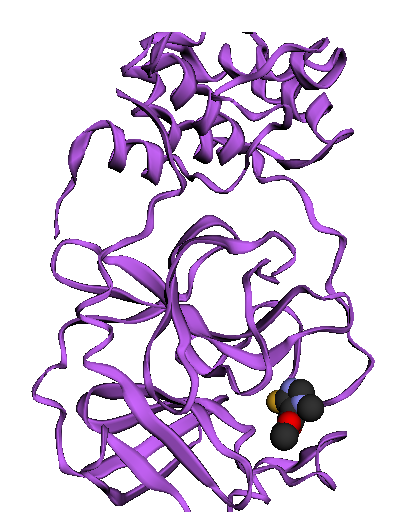 | 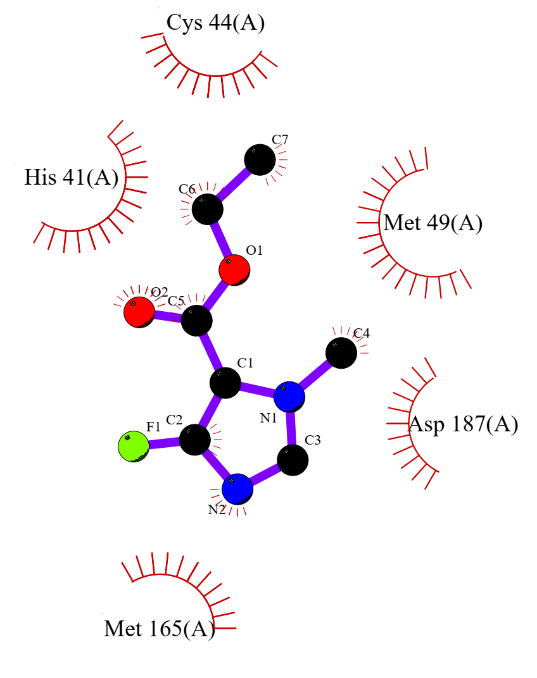 |
| --- | --- |
| **Ethyl 4-fluoro-1-methyl-1H-imidazole-5-carboxylate** | |

| 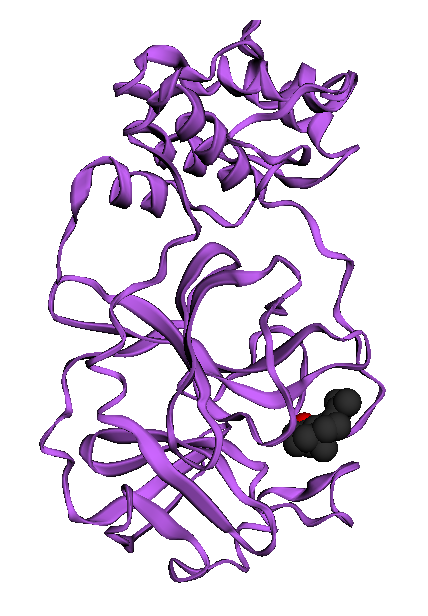 | 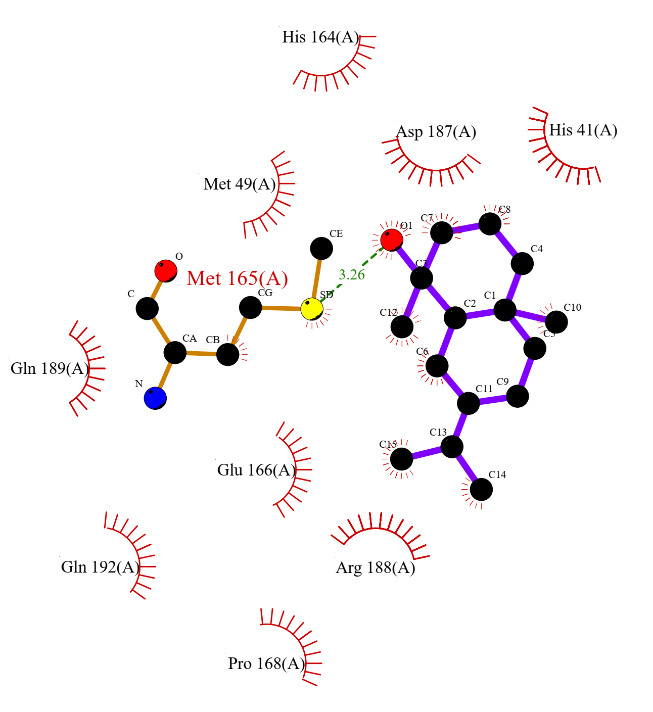 |
| --- | --- |
| **Juniper camphor** | |

| 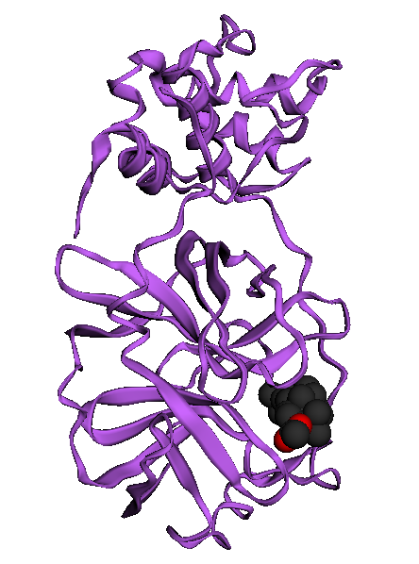 | 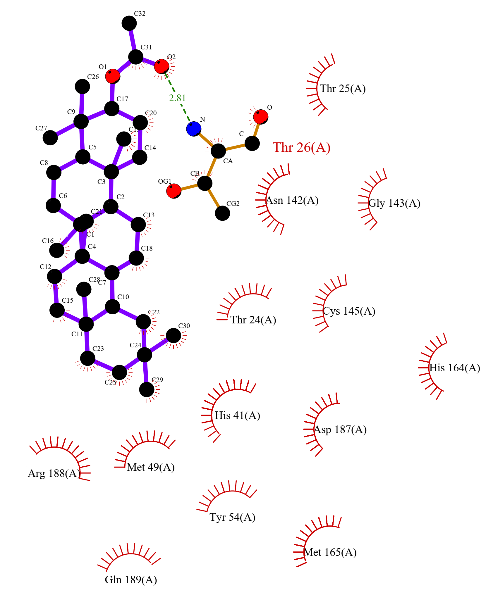 |
| --- | --- |
| **Olean-12-en-3-ol, acetate, (3.beta.)-** | |

| 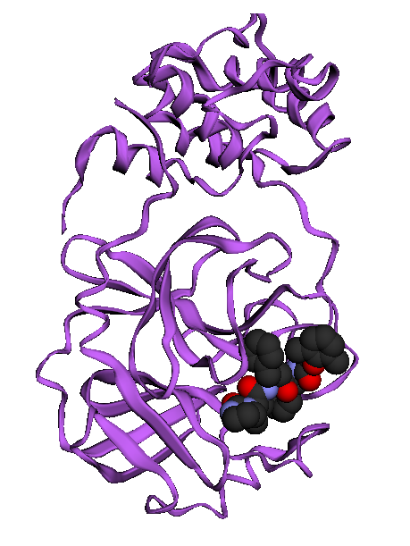 | 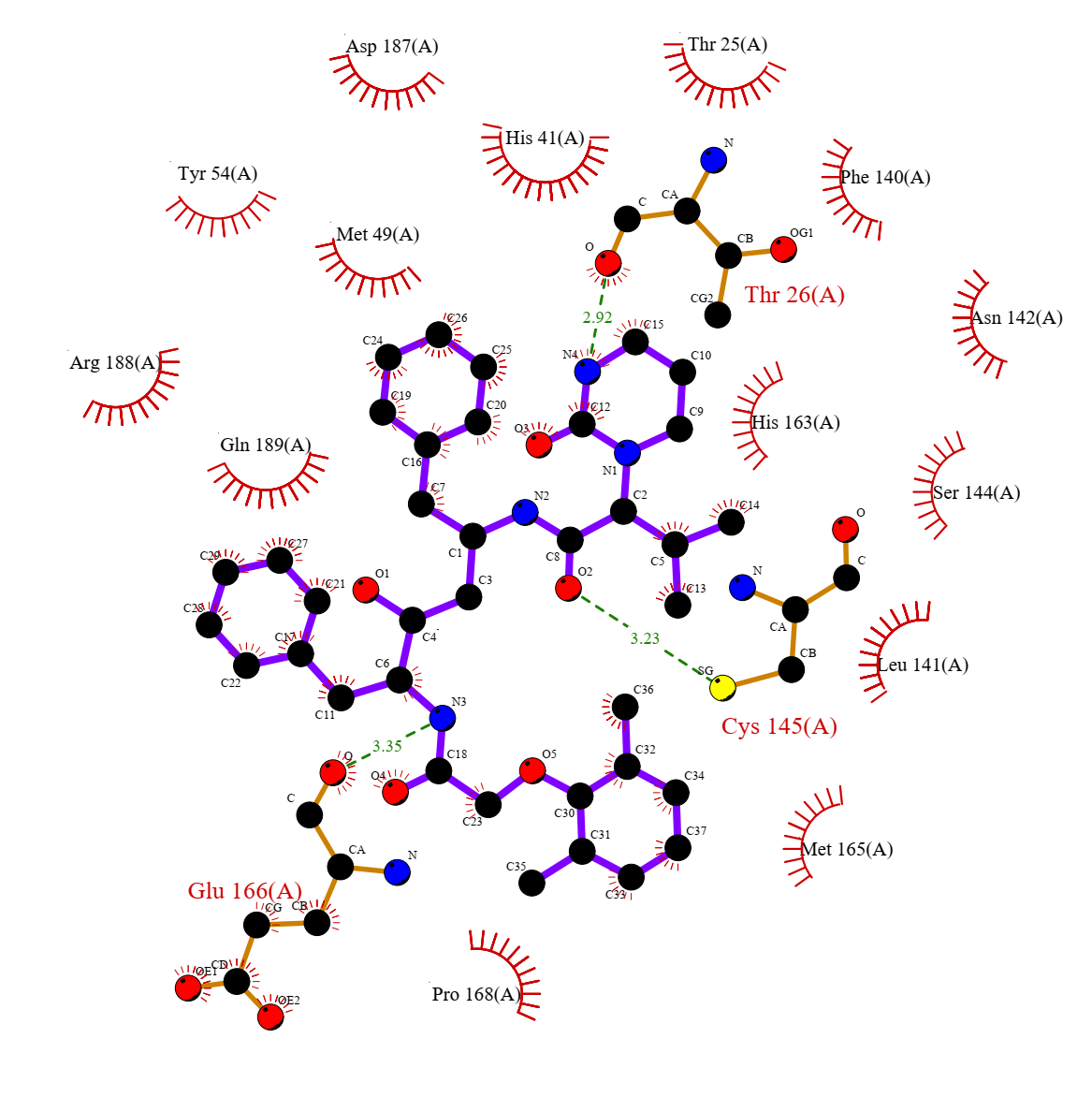 |
| --- | --- |
| **Lopinavir** | |

## FIGURE S1 Visualization of potential interaction by using PDBsum (http://www.ebi.ac.uk/pdbsum) server.

## Supplementary Tables

**TABLE S1** Potential interaction between each ligand molecule and the protein residues calculated through PDBsum (http://www.ebi.ac.uk/pdbsum) server. Red mark indicates the best interactions.

| **Ligand name** | **PubChem ID** | **H-bond** | **Non-bonded** |
| --- | --- | --- | --- |
| Juniper camphor | 521214 | Met165 | **His41 (7)**; Met49; His164; Met165 (3); Glu166; Pro168; Asp187 (3); Arg188 (6); Gln189 (2); Gln192 |
| Ethyl 4-fluoro-1-methyl-1H-imidazole-5-carboxylate | 534521 | - | **His41 (6)**; Cys44 (3); Met49 (2); Tyr54; Met165 (2); Asp187 (5); Arg188 |
| Bicyclo[4.3.0]nonane, 1 isopropenyl-4,5-dimethyl-5-phenylsulfonylmethyl | 595772 | - | Thr25; Thr26; **His41 (6)**; Met49 (8); Tyr54; Asn142; Gly143 (4); Cys145; His164; Met165 (4); Asp187 (7); Arg188 (6); Gln189 (3) |
| Olean-12-en-3-ol, acetate, (3.beta.)- | 91746489 | Thr26 | Thr24 (2); Thr25 (2); Thr26 (5); **His41 (10)**; Met49 (3); Tyr54; Asn142 (6); Gly143; **Cys145**; His164 (3); Met165 (6); Asp187 (6); Arg188 (3); Gln189 |
| Lopinavir | 92727 | Thr26, **Cys145**, Glu166 | Thr25 (4); Thr26 (4); **His41 (9)**; Met49 (3); Tyr54; Phe140 (2); Leu141 (5); Asn142 (3); Ser144; His163; Met165; Glu166 (16); Pro168 (3); Asp187 (5); Arg188 (5); Gln189 (5); Gln189 |
